# Knowledge of cardiovascular disease risk factors and practice of primary prevention of cardiovascular disease by Community Pharmacists in Nigeria: a cross-sectional study

**DOI:** 10.1007/s11096-018-0744-3

**Published:** 2018-11-26

**Authors:** Casmir E. Amadi, Folasade O. Lawal, Amam C. Mbakwem, Jayne N. Ajuluchukwu, David A. Oke

**Affiliations:** 1 Department of Medicine, College of Medicine, University of Lagos, Nigeria, Lagos, Nigeria; 2 Victory Drugs Ltd, Festac, Lagos, Nigeria

**Keywords:** Community Pharmacists, CVD, Hypertension, Primary prevention, Nigeria

## Abstract

*Background* Studies in international literature have shown that Community Pharmacists can make considerable impact in controlling cardiovascular disease risk factors, especially hypertension. In Nigeria, there are no studies on the knowledge of CVD risk factors by Community Pharmacists and their practice of primary prevention. *Objective* To assess the knowledge of CVD risk factors and practice of primary prevention of CVD amongst Nigerian community pharmacists. *Setting* Community Pharmacists in Lagos, Nigeria. *Methods* This cross-sectional study involved 168 Community Pharmacists. Their knowledge of CVD risk factors was assessed with the Heart Disease Fact Questionnaire. Their opportunistic screening practices for CVD risk factors (primary prevention) were also assessed. *Main outcome measures* Knowledge of CVD risk factors and practice of primary CVD prevention. *Results* The mean age of the participating pharmacists was 41.7 (± 11.2) years and 87 (51.8%) of them were males. The median number of years of practice was 9.0 (3–15) years. Mean knowledge score was 22.1 (± 3.0) with 154 (91.7%) of the subjects scoring above 70%. An average of 95.5% of the participants correctly identified hypertension, smoking, dyslipidaemia, obesity, physical inactivity and diabetes as CVD risk factors. Eighty-one (48.2%) had good practice of primary CVD prevention. Conclusion: This study shows that Community Pharmacists in Nigeria have very good knowledge level of CVD risk factors and almost 50% of them practised primary prevention of CVD.

## Impacts on practice

- Because the Nigerian pharmacists in Nigeria have a very good knowledge of cardiovascular risk factors and have the skills to practice primary prevention, it is possible to organise a pharmacists-led mass-screening for cardiovascular disease in Nigeria.
- Continuous training and support in knowledge of cardiovascular disease and prevention practice must be essential components of any government policy that seeks to improve the effectiveness of the primary prevention of CVD in Nigeria.

## Introduction

Cardiovascular disease (CVD) is a pre-eminent but preventable cause of death globally, accounting for an estimated 29% of all deaths [1]. The relative contribution of CVD to the burden of disease remains high in low, middle and high income countries of the world [1]. Interestingly over 80% of the global burden of CVD is borne by the Low and Middle Income Countries (LMICs) in the face of their traditional high prevalence of infectious diseases and poverty-related morbidities and mortalities, constituting a double burden of disease profile [2, 3]. The rising prevalence of CVD in these LMICs is driven by rapid urbanization and its corollary of westernization of lifestyles viz increased consumption of saturated fats and sugars, high salt intake, increasing physical inactivity, smoking and unhealthy use of alcohol [4]. These risky behaviours predispose to the development of the metabolic risk factors for CVD such as obesity, hypertension, diabetes and dyslipidaemia [5]. In Nigeria CVD risk factors are highly prevalent. A recent meta-analysis showed that about 28.9% of adult Nigerians are hypertensive [6]. Similarly, the prevalence of diabetes is between 5 and 10% [7, 8] and dyslipidaemia 60.5% [9]. Prevalence of obesity, smoking and sedentary living is on the increase also [9–11]. Research data provides evidence that there is a rising trend of CVD events and death across Nigeria [12, 13].

Cardiovascular disease is preventable through effective control and management of its risk factors. Data from researches provide sufficient evidence to suggest that the primary prevention of CVD represents a cost-effective approach to reducing this burden [14].

Because patients have difficulties accessing primary-care physicians and health-care costs are rapidly rising, greater use of community-based models of care has been proposed [15]. Among these models is the greater integration of the pharmacist as a provider of health services and member of the health-care team. Community pharmacists are highly accessible health-care professionals in most communities and have demonstrated an effective role in the management of several of these risk factors [16–18]. Their expertise in medicines management, adherence screening and management, and a growing role in health promotion has resulted in several studies demonstrating effective pharmacist involvement with multiple CVD risk factor management [19, 20].

With rising prevalence of CVD risk factors and CVD events and on a background of rising healthcare cost in Nigeria, Community Pharmacists may indeed have a role in stemming this tide. They, being community dwelling, are usually the first point of call for patients and appear more accessible and affordable to them. However, there is dearth of data on the knowledge and practice of primary prevention of CVD by this group of healthcare professionals in Nigeria.

### Aim of the study

This study was designed to assess the knowledge of CVD risk factors and practice of primary prevention of CVD by Community Pharmacists in Lagos, Nigeria.

### Ethics approval

Ethics approval for the study was obtained from the Health Research Ethics Committee of the Lagos University Teaching Hospital, Nigeria.

## Methods

This was a cross sectional study. There were 800 registered Community Pharmacists in Lagos at the time of this study and they met monthly in twenty-two zones in the three senatorial districts of Lagos under the aegis of Lagos State Association of Community Pharmacists. Six zones (two from each of the three senatorial districts) were randomly selected for this study. The selection was based on the population density of the zones.

A sample size of 157 was calculated with the formula used when the survey population is less than 10,000 [21]. Allowing for an attrition rate of 20% the final sample size was 190. The leadership of the Association was approached for permission to carry out the study on their members. Their members were invited to participate in the study through a letter written by the researcher. The letter was read out to them by the Secretary of the Association in the plenary session of one of their monthly meetings, explaining the rationale for the study. To further enhance participation rate a copy of this letter was also pasted on the Association’s Notice Boards in the six zones selected for the members to read. We aimed at recruiting 32 pharmacists from each of the six zones. One hundred and ninety pharmacists agreed to participate in the study but only 168 completed and returned their questionnaire giving a response rate of 88.4%. Trained research personnel were used to administer a pretested, structured self-administered questionnaire to the consenting pharmacists during their monthly zonal meetings. Completing the questionnaire took an average of 20 min. The entire period of the study lasted for 12 weeks. The questionnaire was initially pretested among a group of twenty Community Pharmacists in Lagos. Their responses were used to fine tune the final version used for the study.

The questionnaire consisted of four sections. Section one comprised the socio-demographic information of the participants while section two assessed their knowledge of CVD risk factors using the widely validated Heart Disease Fact Questionnaire (HDFQ). The HDFQ is a 25-item measure of heart disease knowledge which evaluates of knowledge of risk factors for heart disease, the link between diabetes and heart disease and how to reduce the risk of heart disease in the diabetic population [22, 23]. It has been used in other populations with reliable test–retest reliability, internal consistency and satisfactory discriminant validity [22] and has also been used among staff members of a university in Nigeria in 2015 [24]. Responses were ‘True’, ‘false’ or ‘I don’t know’. Scores were summed up and total score greater than 20 indicated good knowledge. Scores were also expressed in percentages. Score of < 50% was classified as low level of knowledge, between 50 and 69% as moderate level of knowledge and HDFQ score > 70% as good level of knowledge. Questions that less than 70% of the respondents answered correctly were deemed unsatisfactory. Section three assessed the participants’ knowledge of the diagnostic cut-offs for hypertension, diabetes, dyslipidaemia and obesity while section four assessed their practice of primary prevention of CVD and comprised seven questions viz availability of functional sphygmomanometers, glucometers, routine measurement of blood pressure for their clients, routine measurement of BMI/waist circumference, awareness of any guidelines on hypertension, how frequently they advised their hypertensive and diabetic clients to check their cholesterol profile and how frequently they counselled their clients on healthy lifestyle choices in their Practices. Each of these questions was scored 1 point if the participant responded to them in the affirmative and zero if otherwise. Score < 2 was regarded as poor practice, 3–4 as fair while greater than 4 as good. The questions in sections 3 and 4 were included to make the assessment of CVD knowledge more stringent and rigorous since the participants, being healthcare professionals, might find the HDFQ easier than the lay public whom it was originally designed for.

### Statistical analysis

Data was analysed with SPSS software version 21.0. Numerical data was expressed as mean (SD) while categorical data was expressed as percentages. Chi-square test was used to compare proportions. A *p* value < 0.05 indicated statistical significance.

## Results

### General characteristics of the subjects

The mean age of the study population was 41.7 (± 11.2) years while 87 (51.8%) were males. The median number of years of practice was 9.0 (3.0–15.0) years with 53 (31.5%) having various postgraduate qualifications. Majority 87 (51.7%) had their practices located in the urban areas. One hundred and fifty-six (92.8%) saw at least 10 hypertensives monthly in their practices while 144 (85.7%) and 85 (50.6%) saw a minimum of 10 diabetics and smokers respectively every month in their practices. The rest of their general characteristics is shown in Table 1.

**Table 1:** General characteristics of respondents

Variable	Frequency (%)
Age of respondents (years)	
≤ 30	36 (21.4)
31–45	71 (42.3)
46–60	51 (30.4)
> 60	10 (6.0)
Mean (± SD)	
41.73 (± 11.2) years	
Gender	
Male	87 (51.8)
Female	81 (48.2)
Marital status	
Single	37 (22.0)
Married	131 (78.0)
Additional qualification	
None	115 (68.5)
MSc	20 (11.9)
Diploma/Cert	14 (8.3)
Others	19 (11.3)
Number of years of practice	
≤ 5	64 (38.1)
6–10	35 (20.8)
> 10	69 (41.1)
Median (Q1, Q3)	9.0 (3.0, 15.0)
Location of practice	
Urban	86 (51.2)
Semi/urban	73 (43.4)
Rural	9 (5.4)
Average number of hypertensives seen per month	
< 10	12 (7.1)
10–20	44 (26.2)
> 20	112 (66.7)
Average number of diabetics seen per month	
<10	24 (14.3)
11–20	72 (42.9)
> 20	72 (42.9)
Number of smokers seen per month	
<10	83 (49.4)
10–20	62 (36.9)
> 20	23 (13.7)

### Knowledge of CVD risk factors

Stroke was correctly identified as an example of a CVD by 73 (47.4%) of the respondents while the same number of respondents wrongly identified hypertension as a CVD. On the other hand 136 (81.4%) of the pharmacists correctly identified smoking as a CVD risk factor. Table 2 shows the number of the participants who correctly identified the diagnostic cut-off points for hypertension, diabetes, obesity with BMI, abdominal obesity and treatable dyslipidaemia.

**Table 2:** Knowledge of diagnostic cut-off for common CVD risk factors

Risk factor	Cut-off	Frequency (%)
Hypertension	^a^BP ≥ 140/90 mmHg	97 (57.7)
	BP > 130/95 mmHg	19 (11.3)
	BP > 120/80 mmHg	37 (22.0)
	BP > 150/90 mmHg	1 (0.6)
	Unknown	14 (8.3)
Diabetes	^a^FBS ≥ 126 mg/dl	53 (31.5)
	FBS > 140 mg/dl	23 (13.7)
	FBS > 110 mg/dl	67 (39.9)
	Unknown	25 (14.9)
Obesity	^a^BMI ≥ 30 kg/m^2^	43 (25.6)
	BMI > 35 kg/m^2^	32 (19.0)
	BMI > 25 kg/m^2^	52 (31.0)
	Unknown	41 (24.4)
Abdominal obesity (male)	^a^WC > 102 cm	38 (22.6)
	> 88 cm	14 (8.3)
	> 90 cm	32 (19.0)
	Unknown	84 (50.0)
Abdominal obesity (female)	> 102 cm	20 (11.9)
	^a^> 88 cm	43 (25.6)
	> 90 cm	17 (10.1)
	Unknown	88 (52.4)
Hypercholesterolaemia	^a^Tc ≥ 240 mg/dl	22 (13.1)
	Tc 220 mg/dl	18 (10.7)
	Tc 200 mg/dl	37 (22.0)
	Tc 190 mg/dl	15 (8.9)
	Unknown	76 (45.2)

*BP* blood pressure, *FBG* fasting blood glucose, *BMI* Body Mass Index, *WC* waist circumference, *Tc* total cholesterol

^a^Correct answer

### Assessment of knowledge of CVD risk factors

**Fig. 1:**
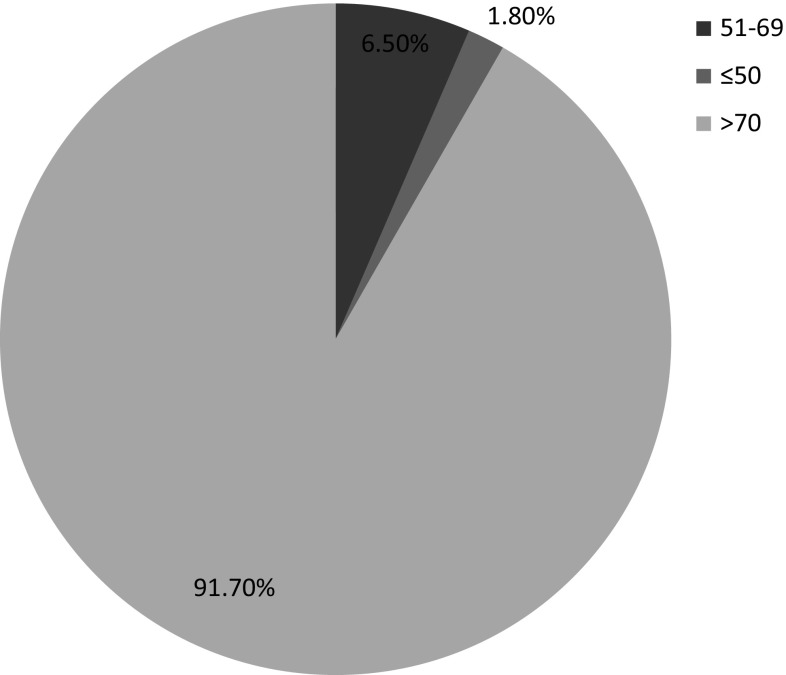
Pie chart showing the distribution of the HDFQ scores of the respondents

**Fig. 2:**
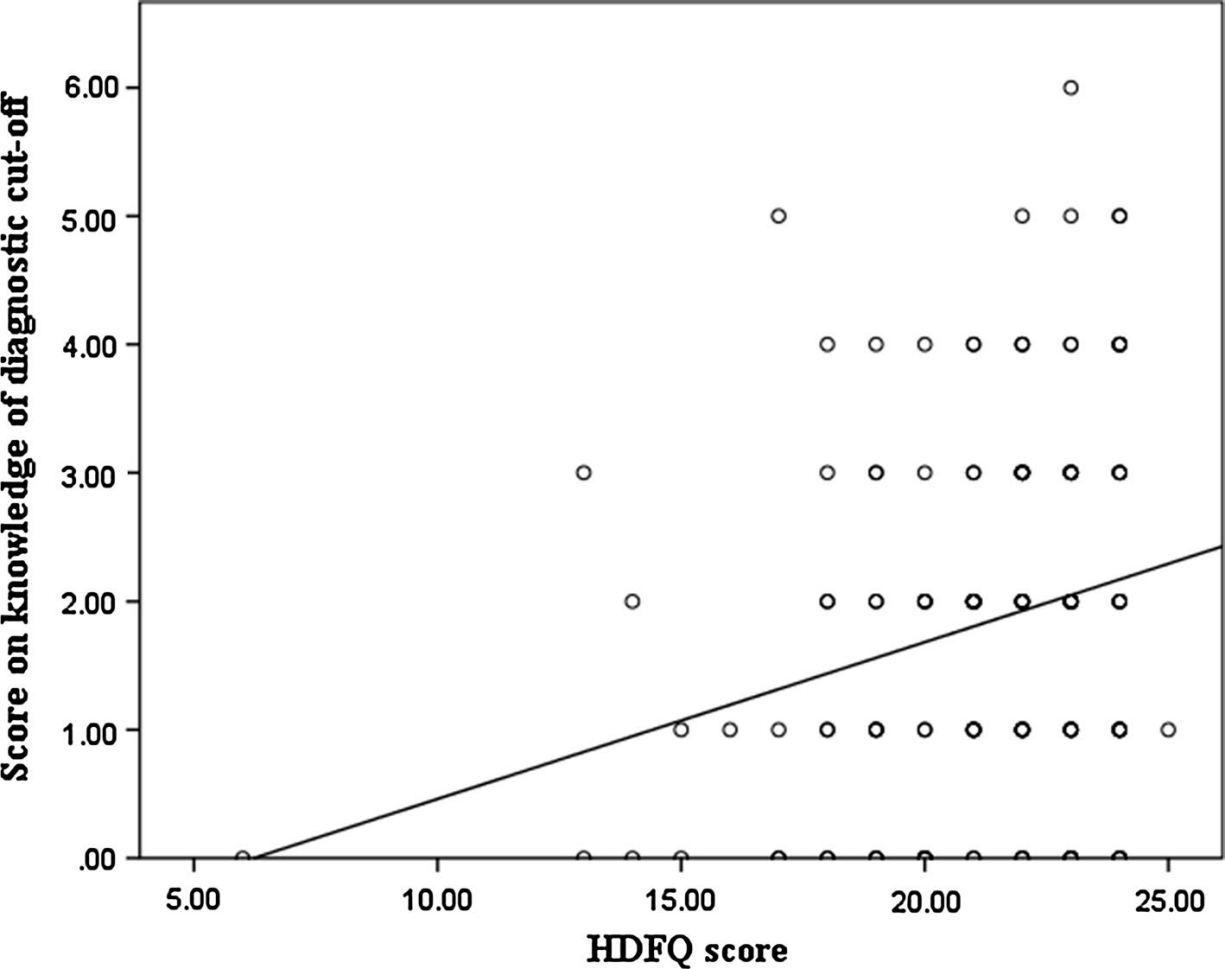
Scatter plot showing the relationship between HDFQ scores and knowledge of diagnostic cut off for common CVD risk factors. Spearman correlation coefficient: 0.317; *p* = 0.045

Figure 1 shows the frequency scores of the HDFQ while Table 3 shows the responses of the participants to the items on the HDFQ. About 95.5% of the participants could identify the traditional risk factors for CVD viz hypertension (97.6%), smoking (97%), dyslipidaemia (97%), obesity (96.4%), physical inactivity (95.2%) and diabetes (91.7%), indicating high knowledge. They were also very knowledgeable of the impact of managing/controlling these risk factors in the development of heart disease and the relationship between diabetes and other CVD risk factors as they affect the risk of development of heart disease. However, participants displayed low knowledge of level High Density Lipoprotein cholesterol (HDL-c) in diabetes and females with diabetes having a higher risk of development of heart disease. Table 4 shows the association between the HDFQ scores and some of the socio-demographic characteristics of the participants. There was no relationship between age, gender, location of practice, duration of practice, possession of additional qualifications with HDFQ scores. Figure 2 shows the linear relationship between the HDFQ scores and knowledge of the diagnostic cut-offs for the CVD risk factors. The higher the HDFQ scores the more likely the pharmacists would correctly identify the diagnostic cut off (*p* = 0.045).

**Table 3:** Correct responses to HDFQ questionnaire

Questions	Frequency (%)
A person always knows when they have heart disease	142 (84.5)
If someone has a family history of heart disease, he/she is at risk for developing heart disease	157 (93.5)
The older a person is, the greater their risk of having heart disease	151 (89.9)
Smoking is a risk factor for heart disease	163 (97.0)
A person who stops smoking will lower their risk of heart disease	159 (94.6)
High blood pressure is a risk factor for heart disease	164 (97.6)
Keeping blood pressure under control will reduce a person’s risk for developing heart disease	162 (96.4)
High cholesterol is a risk factor for developing heart disease	163 (97.0)
Eating fatty foods does not affect blood cholesterol	157 (93.5)
If someone’s good cholesterol (HDL) is high he/she is at risk for heart disease	138 (82.1)
If someone’s bad cholesterol (LDL) is high he/she is at risk for heart disease	155 (92.3)
Being overweight increases a person’s risk for heart disease	162 (96.4)
Regular physical activity will lower a person’s chance of getting heart disease	160 (95.2)
Only exercising at a gym or in an exercise class will lower a person’s chance of developing heart disease	146 (86.9)
Walking and gardening are considered exercise that will help lower a person’s chance of developing heart disease	140 (83.3)
Diabetes is a risk factor for developing heart disease	154 (91.7)
High blood sugar puts a strain on the heart	131 (78.0)
If someone’s blood sugar is high over several months it can cause his/her cholesterol level to go up and increase his/her risk of heart disease	108 (64.3)
A person who has diabetes can reduce his/her risk of developing heart disease if he/she keeps his/her blood sugar level under control	153 (91.1)
Person with diabetes rarely have high cholesterol	130 (77.4)
If a person has diabetes, keeping his/her cholesterol under control will help lower his/her chance of having heart disease	145 (86.3)
People with diabetes tend to have low HDL (good) cholesterol	49 (29.2)
A person who has diabetes can reduce his/her risk of developing heart disease if he/she keeps his/her blood pressure under control	150 (89.3)
A person who has diabetes can reduce his/her risk of developing heart disease if he/she keeps his/her weight under control	153 (91.1)
Men with diabetes have a higher risk of heart disease than women with diabetes	37 (22.0)

**Table 4:** Association between HDFQ score category and socio-demographic characteristics

Characteristics	HDFQ score category		X^2^	*p* value
	> 70	≤ 70		
*Age of respondents (years)*			2.184	0.534
≤ 30	31 (86.1)	5 (13.9)		
31–45	67 (94.4)	4 (5.6)		
46–60	47 (92.2)	4 (7.8)		
> 60	9 (90.0)	1 (10.0)		
*Gender*			0.176	0.675
Male	79 (90.9)	8 (9.2)		
Female	75 (92.6)	6 (7.4)		
*Marital status*			0.003	0.955
Single	34 (91.9)	3 (8.1)		
Married	120 (91.6)	11 (8.4)		
*Additional qualification*			1.127	0.771
None	107 (93.0)	8 (7.0)		
MSc	18 (90.0)	2 (10.0)		
Diploma/Cert	12 (85.7)	2 (14.3)		
Others	17 (89.5)	2 (10.0)		
*Number of years of practice*			1.111	0.574
≤ 5	58 (90.6)	6 (9.4)		
6–10	31 (88.6)	4 (11.4)		
> 10	65 (94.2)	4 (5.8)		
Median (Q1, Q3)	9.0 (3.0, 15.3)	6.0 (1.8, 14.8)		
*Location of practice*			5.096	0.078
Urban	82 (95.3)	4 (4.7)		
Semi/urban	63 (86.3)	10 (13.7)		
Rural	8 (100.0)	0 (0.0)

### Practice of primary prevention of CVD

**Fig. 3:**
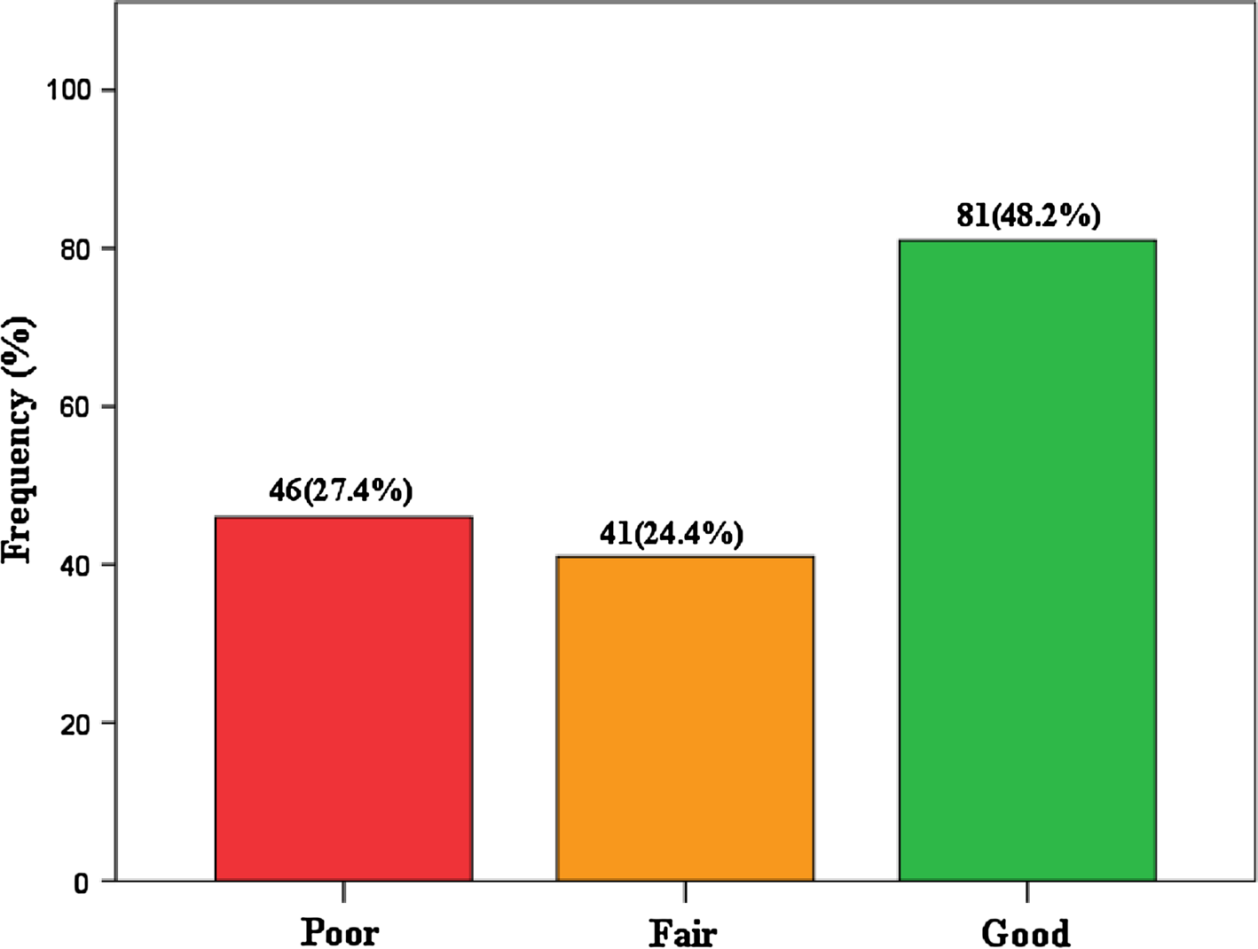
Bar chart showing scores on the practice of primary prevention of CVD among the participants

One hundred and sixty of the pharmacists (95.2%) indicated that they had functional sphygmomanometers while 139 (82.7%) routinely measured the blood pressure of most clients who consulted them for a broad range of reasons. Details of pharmacists’ practice of primary prevention of CVD are shown in Table 5. With regards to knowledge of any guidelines for hypertension 73 (43.4%) were aware of the existence of guidelines on hypertension but none was able to mention any including the JNC VII guideline, which was the operational hypertension guideline in Nigeria as at the time of this study. Figure 3 the shows frequency of the scores on the practice of primary prevention of CVD. Eighty-one (48.2%) of them had good primary CVD prevention practice score. Table 6 shows an association between the HDFQ scores and practice of primary prevention of CVD. Those with HDFQ scores greater than 70% had higher primary CVD prevention scores (*p* = 0.026).

**Table 5:** Practice Primary Prevention of CVD among the Respondents

Practice	Frequency	Percentage
Availability Functional BP Sphygmomanometers	160	95.2
Routinely measure the BP of hypertensive client	139	82.7
Functional glucometer to measure blood glucose	122	72.6
Routinely measure BMI/waist circumference	48	28.6
Awareness of guideline on hypertension	73	43.5
*Frequency of informing hypertensives or diabetics to check their cholesterol level*		
Very often	84	50.0
Sometimes	54	32.1
Rarely	20	11.9
Never	10	6.0
*Frequency of advising hypertensives or diabetics on lifestyle management*		
Very often	116	69.0
Sometimes	38	22.6
Rarely	4	2.4
Never	10	6.0

**Table 6:** Association between HDFQ score grade and primary prevention practices

Primary prevention practice	HDFQ Score grade		X^2^	p-value
	> 70	≤ 70		
Poor	38 (82.6)	8 (17.3)	7.269	0.026*
Fair	38 (92.7)	3 (7.3)		
Good	78 (93.3)	3 (3.7)		

*Significant

## Discussion

This study, first of its kind in Nigeria (to the best knowledge of the authors), shows that Community Pharmacists in Lagos, Nigeria have very good knowledge of CVD risk factors using the HDFQ instrument. This contrasts with the findings from a similar study done amongst staff members of a tertiary institution in South West Nigeria where 19.9% of the participants had good knowledge of CVD risk factors [24]. Wagner et al. [23], using the same instrument, also demonstrated low knowledge of CVD risk in a population of Spanish speaking diabetics in Puerto Rico. The higher level of knowledge observed in our study was not surprising since the studied population comprised healthcare professionals who should be knowledgeable about CVD. In our study the knowledge of these risk factors was independent of age, gender, location of practice, duration of practice and possession of additional qualifications and corroborates findings from similar studies [23, 24]. This high level of knowledge displayed by the participants in this study may provide a basis for a feasibility study on the effectiveness of Community Pharmacists in CVD risk factor screening in Nigeria.

Our study further demonstrated that the participants displayed good knowledge of both the behavioural (physical inactivity, smoking and unhealthy diets) and biologic (hypertension, diabetes, obesity and dyslipidaemia) risk factors for CVD. However in relative terms knowledge of diabetes and measures of obesity was lower. This was reflected in the lower of number of participants who correctly identified the diagnostic cut-offs for diabetes and abdominal obesity compared to hypertension and also the lower number of participants who screened clients for diabetes and obesity in their practices compared to hypertension. The reason for this might be might be due to the higher prevalence of hypertension compared to diabetes in the general Nigerian population [6, 8]. Secondly the invasive nature of screening for diabetes with a glucometer could be a demotivation for them and their clients. For obesity the technicalities for measuring parameters for obesity (waist circumference and body mass index) accurately may be exerting on the pharmacists in addition to time and space constraints. These may discourage them from screening for obesity. A similar study in Australia found availability of space a determinant of screening for obesity [25].

Our study also found that majority of the pharmacists were involved in primary prevention of CVD through screening for risk factors and counselling on the importance of healthy lifestyle choices. Hypertension screening was more commonly done than for diabetes and obesity. This might also be explained by the reasons adduced above. Joyce et al. [25], studying the practice of primary prevention of CVD among Community Pharmacists in Australia, found that hypertension was the most commonly screened risk factor, similar to what our study demonstrated.

Studies from various parts of the world have documented the feasibility of using Community Pharmacists in CVD screening in their practices [26–28]. These screening programmes were mostly opportunistic in nature and involved both healthy and ill individuals. This feasibility compares well with studies that have demonstrated the feasibility of CVD screening by other health professionals [29, 30]. Some of these screening efforts reported prevalence of CVD risk factors similar to national prevalence and were also able to detect previously undiagnosed risk factors [28, 31]. Rohla et al. [31], in a cross-sectional study involving 184 pharmacies in Lower Austria, found an overall prevalence of CVD risk factors similar to reported prevalence in national surveys with about 30% of the cases being newly diagnosed. In addition public uptake of these screening programmes was positive [32, 33]. Furthermore in some of these studies identified cases were referred to the physicians for further evaluation and definitive management suggesting possible opportunities for a collaborative care model involving the physicians and the pharmacists [28, 31]. In summary, these studies demonstrate that CVD screening by Community Pharmacists is feasible, accessible and acceptable to the public and might be an effective alternative to public screening.

The success recorded above may be attributable to a host of factors. First, Community Pharmacists are located within the community and thus are usually the first point of contact and in some cases the only point of contact for patients seeking the services of a health-care provider without an appointment [18, 34]. In addition, they have extended hours of service which further increases accessibility to care. Being community dwelling, they build informal and genial relationships with members of their host communities, engendering their trust and loyalty. This places them in a vintage position to implement primary prevention strategies for CVD and collaborate with the physician in ensuring continuity of care [18, 34]. In some settings healthcare delivery service by them appears relatively cheaper and more cost effective for the patient [34].

Despite these benefits, some factors have been identified that could potentially hinder the ability and effectiveness of Community Pharmacists in CVD screening. In climes where pharmacists have been involved in CVD screening some barriers have been identified that limit their effectiveness. These include time constraints, limited physical space to ensure privacy of the patients, need for further training, lack of regulatory policy, remuneration and reimbursement mechanisms and poor uptake of CVD screening services by the public [33, 35–37]. In Nigeria government policy integrating Community Pharmacists fully into the nation’s healthcare delivery system is apparently lacking. They operate primarily as private commercial ventures offering pharmaceutical services that complement the government’s healthcare system. In the absence of a government policy, training and certifying them to render CVD care delivery remains impracticable. By extension the expected collaborative linkage between them and physicians will at best be weak as they would be seen to be offering these services outside the ambit of government policy. This could also affect the public’s confidence in the uptake of such services. Time management constraints, lack of space, lack of training and lack of reimbursements might be potential impediments as in other climes. Most community pharmacies in Nigeria are sole proprietorships, driven by profit. Introduction of CVD care delivery services that will involve personal training to improve competencies, time and physical space resources and which are not recognised by the government nor covered by any reimbursement schemes of the National Health Insurance Program and which the patients may be unwilling to pay for might not be commercially appealing to them and may likely discourage them from venturing into such. Further, there is the issue of perceived ‘turf encroachment’, whereby pharmacists are perceived or act to supplant the role of physicians and compete rather than collaborate with them. This has been reported as another major impediment to the incorporation of pharmacists in mainstream CVD care [18]. In Nigeria, inter-professional rivalry in the health sector is rife, leading to frequent industrial disharmony and friction between the other health workers on one hand and the physicians on the other hand. The issue of ‘turf encroachment’ will be a major consideration in any collaborative healthcare delivery model that will involve the physician and the pharmacist as the latter might rightly or otherwise be seen to usurp or compete rather collaborate with the former. Issues such as ‘ownership of patients’ and remuneration structure might be potential sources of conflicts should this collaboration become operational. This scenario would be made worse in the absence of a government policy and operational guidelines. These are serious issues that may hinder the effectiveness of these health professionals in CVD screening services.

### Limitations

This study has some limitations. The HDFQ used in this study was designed for assessment of knowledge of CVD risk factors in the lay public. Our study population comprised Community Pharmacists who are highly trained healthcare professionals. Thus results from the HDFQ are subject to bias. Secondly some of the questions involved self-report, which could have also introduced recall bias. The size of the population studied was small and this might have affected the results and the conclusions.

## Conclusions

Our study has shown that Community Pharmacists in Nigeria have very good knowledge of CVD risk factors and also practice primary prevention of CVD. Thus assessment of the feasibility of using them for CVD risk factor screening is possible if enabling factors are in place. However, their knowledge of diabetes and obesity needs to be deepened. For them to be effective in the provision of these screening services there should a government policy that will regulate their training, certification, practice for CVD screening and as well as provide a framework of incentivising this service.
